# Boosting Biomass Quantity and Quality by Improved Mixotrophic Culture of the Diatom *Phaeodactylum tricornutum*

**DOI:** 10.3389/fpls.2021.642199

**Published:** 2021-04-09

**Authors:** Valeria Villanova, Dipali Singh, Julien Pagliardini, David Fell, Adeline Le Monnier, Giovanni Finazzi, Mark Poolman

**Affiliations:** ^1^Department of Biological and Environmental Sciences, University of Gothenburg, Gothenburg, Sweden; ^2^Laboratoire de Physiologie Cellulaire et Végétale, Université Grenoble Alpes (UGA), Centre National de la Recherche Scientifique (CNRS), Commissariat á l'Énergie Atomique et aux Énergies Alternatives (CEA), Institut National de Recherche pour l'Agriculture, l'Alimentation et l'Environnement, Interdisciplinary Research Institute of Grenoble, CEA Grenoble, Grenoble, France; ^3^Fermentalg SA, Libourne, France; ^4^Microbes in the Food Chain, Quadram Institute Biosciences, Norwich Research Park, Norwich, United Kingdom; ^5^Cell System Modelling Group, Oxford Brookes University, Oxford, United Kingdom

**Keywords:** genome-scale metabolic model, linear programming, metabolism, mixotrophic growth, diatom, *P. tricornutum*, biomass productivity

## Abstract

Diatoms are photoautotrophic unicellular algae and are among the most abundant, adaptable, and diverse marine phytoplankton. They are extremely interesting not only for their ecological role but also as potential feedstocks for sustainable biofuels and high-value commodities such as omega fatty acids, because of their capacity to accumulate lipids. However, the cultivation of microalgae on an industrial scale requires higher cell densities and lipid accumulation than those found in nature to make the process economically viable. One of the known ways to induce lipid accumulation in *Phaeodactylum tricornutum* is nitrogen deprivation, which comes at the expense of growth inhibition and lower cell density. Thus, alternative ways need to be explored to enhance the lipid production as well as biomass density to make them sustainable at industrial scale. In this study, we have used experimental and metabolic modeling approaches to optimize the media composition, in terms of elemental composition, organic and inorganic carbon sources, and light intensity, that boost both biomass quality and quantity of *P. tricornutum*. Eventually, the optimized conditions were scaled-up to 2 L photobioreactors, where a better system control (temperature, pH, light, aeration/mixing) allowed a further improvement of the biomass capacity of *P. tricornutum* to 12 g/L.

## 1. Introduction

Diatoms are photosynthetic unicellular microalgae that dominate the oceans. Their ability to synthesize lipid as a storage compound makes them a potential source of biofuel and high-value commodities such as omega fatty acids (Hildebrand et al., 2012; d'Ippolito et al., 2015; Wang and Seibert, 2017; Yi et al., 2017; Pudney et al., 2019). They have notably different evolutionary history from that of other photosynthetic eukaryotes such as plants and green algae, and are thought to have arisen from a complex endosymbiotic event, which is ascertained, though donor is not clearly identified (Wilhelm et al., 2006; Armbrust, 2009; Moustafa et al., 2009). Consequently, diatoms have a number of unique biochemical features distinguishing them other photosynthetic eukaryotes.

Unlike other photosynthetic eukaryotes, enzymes associated with the Calvin cycle, in diatoms, are not solely located in the chloroplast; possibly the Calvin cycle enzyme sedoheptulose-bisphosphatase (SBPase) is instead located in the cytosol (Kroth et al., 2008), suggesting that either other chloroplast enzymes compensate for the absence of SBPase, or that the Calvin cycle is distributed between the chloroplast and cytosol. Similarly, enzymes of the oxidative limb of the oxidative pentose phosphate pathway (OPPP) are located in the cytosol, not the plastid. In other photosynthetic eukaryotes, glycolysis follows the well-known Embden-Meyerhof-Parnass (EMP) scheme and is located in the cytosol. The situation in *Phaeodactylum tricornutum* is somewhat different; the cytosolic pathway lacks the enzyme enolase, with the result that the cytosolic pathway is truncated at phosphoglycerate (PGA). However, enolase and pyruvate kinase are found in both the chloroplast and the mitochondrion leading to the possibility of glycolysis being distributed between two or even three subcellular compartments (Liaud et al., 2000; Kroth et al., 2008; Smith et al., 2012). The Entner–Doudoroff (ED) pathway and phosphoketolase pathways, commonly found in prokaryotes, have been reported to be present in *P. tricornutum* (Fabris et al., 2012), though the contribution of these pathways in *P. tricornutum* is not well-known.

Diatoms can produce biomass via different modes of growth: phototrophic and mixotrophic. The former mode simply converts the sunlight energy into reduced carbon via photosynthesis. This can be a cheap way to produce biomass because only sunlight is used as an energy source, but it is often limited by the difficulties in optimizing light penetration, gas diffusion, and temperature control in photobioreactors and race ponds (Chisti, 2007, 2008). While some microalgae can grow heterotrophically via sugar fermentation in the dark, this is not possible in *Phaeodactylum*, which can use sugars in the dark only upon metabolic engineering (Zaslavskaia et al., 2001). On the other hand, *P. tricornutum* can grow mixotrophically, i.e., using simultaneously light and reduced carbon (Garcì et al., 2004; Garcìa et al., 2005, 2006, 2013; Liu et al., 2009a). This mode of cultivation represents an interesting alternative to phototrophic growth, because mixotrophically grown algae have, in principle, a lower requirement for optimum light penetration, and can make use of cheap and easily available carbon sources, like glycerol, leading to a high biomass productivity (Garcì et al., 2004; Garcìa et al., 2005, 2006, 2013).

Nitrogen and phosphorus (after carbon) are often the most important macronutrients required for the growth of microalgae (Wijffels and Barbosa, 2010) and the deprivation of these elements has been reported to trigger TAG accumulation in *Phaeodactylum* (Abida et al., 2015). However, deprivation of these elements also restricts growth, and thus biomass quantity. Micronutrients are essential for algal growth because of their role as cofactors of key enzymes (Morel et al., 2003; Merchant and Helmann, 2012; Blaby-Haas and Merchant, 2017). For instance, iron is a redox active metal present in several metalloproteins involved in photosynthesis, respiration, and nitrogen assimilation and hence is very important in phototrophs (Moore et al., 2013). Manganese also plays a very important role (Sunda and Huntsman, 1996). Indeed, the manganese is involved in such vital processes of phototrophic organisms as the oxidation of water done by the PSII complex, and the conversion of superoxide radical into molecular oxygen (O_2_) and hydrogen peroxide (Blaby-Haas and Merchant, 2017; Saavedra et al., 2018). Zinc is also essential, as shown by the examples of the green alga *Chlamydomonas reinhardtii*, where it impacts CO_2_ assimilation and Cu homeostasis (Malasarn et al., 2013), and of the diatom *T. weissflogii*, where it seems to be associated with carbonic anhydrase (CA) regulating the rate of carbon uptake and fixation (Morel et al., 1994). Copper has also an effect on diatoms' growth, possibly because this element is involved in the regulation of Fe uptake (Annett et al., 2008) and, in some cases, in the substitution of Fe-containing enzymes by Cu-containing enzymes (e.g., plastocyanin in *T. oceanica*) (Peers and Price, 2006). However, too elevated concentrations of these elements may result in oxidative damage or in decrease of growth rate of cells (Choudhary et al., 2007).

The f/2 medium by Guillard (1975) (half the concentration of the original f medium designed by Guillard and Ryther, 1962) is one of the earliest designed and a widely used enriched seawater medium for growing coastal marine algae, especially diatoms. It is composed of filtered natural seawater, trace elements, and vitamins. However, the use of natural seawater can result in seasonal variability and difference in quality, which led to design of artificial seawater medium to provide better quality control. Enriched seawater artificial seawater (ESAW) (Harrison et al., 1980) is another extensively used medium for microalgae physiological study. It composition is constant and its use is not detrimental for the algae. Some microalgae can reach a higher biomass yield in ESAW than in the f/2 medium. The ESAW medium was later modified by Berges et al. (2001), for salts and metals, leading to improved marine algal growth. For these reasons, we decided to use the modified ESAW as the starting medium in this study.

Light is another important parameter to consider for the optimization of phototrophic and mixotrophic algal cultivation (Falkowski and Owens, 1980; Rashid et al., 2015). Increasing the light intensity usually tends to increment the algal growth rate up to the light saturation level. However, when light becomes oversaturating, it can lead to the formation of harmful products (i.e., ROS) and, hence, decrease algal biomass productivity (Richmond, 2000). To avoid this phenomenon of photoinhibition, light intensity has to be optimized in relation to cell density, so that the culture does not become light limited or photodamaged (Eriksen, 2008; Zhu, 2015; Sivakaminathan et al., 2018). The optimization of medium composition for micro- and macronutrients, carbons source and light intensity is a crucial step for enhancing the biomass productivity and lipid concentration of the selected microalgae.

The genome-encoded metabolic potential, as a genome-scale metabolic model (GSM), has been previously exploited to define optimal conditions, design growth media, and predict metabolic fluxes under different genetic and environmental conditions (Kim et al., 2017; Pan and Reed, 2018; Gu et al., 2019; Ong et al., 2020; Tejera et al., 2020). Its analysis enables an investigation of metabolic behavior of the whole system and has been utilized to inform experimental design and to provide a rationale for experimental observations (McCloskey et al., 2013; Villanova et al., 2017; Mishra et al., 2018; van der Ark et al., 2018).

In this work, we have used a genome-scale metabolic model and an experimental approach to test the role of inorganic and organic carbon (glycerol) sources in *P. tricornutum* biomass productivity and optimized the elemental composition of macro- and micronutrients in the ESAW medium (Berges et al., 2001) in order to enhance both biomass quantity and quality. The optimized medium composition was used to scale-up *P. tricornutum* culture to a 2-L photobioreactor where light intensity was further optimized for cell density enhancement. Using our enhanced optimized media, biomass and fatty acid production in *P. tricornutum* increased by a factor of about 9 and 45, respectively.

## 2. Materials and Methods

### 2.1. Strains, Growth Media, and Culture Conditions

Axenic cultures of *P. tricornutum* (Pt1, CCAP 1055/3, Martino et al., 2007) were grown in a 250 mL Erlenmeyer flask in an artificial seawater ESAW (Berges et al., 2001) supplemented with extra NaNO_3_ and NaH_2_PO_4_ to reach a final concentration of 0.47 g/L N and 0.03 g/L P (this medium will be referred as E10 from now on). Cells were grown in a chamber at 20°C, 40 μE m^−2^ s^−1^ irradiance with a led light for 12-h light/12-h dark photoperiod and shaking at 100 rpm. For mixotrophic growth experiments, filter sterilized glycerol was added at a final concentration of 4.6 g/L (E10+GLY).

To monitor algal growth, samples were taken daily (in the end of the light period) and growth was estimated by (i) calculating dry cell weight (DCW) and (ii) measuring the optical density at 750 nm using a double beam UV/visible spectrophotometer from Thermo Fisher Scientific. In the latter case, cell concentration was evaluated from a calibration curve obtained by the correlation of the absorbance at 750 nm and dry weight (Supplementary File 1). The growth profiles in the different media were compared as a function of Ln(C/C0), where C is the biomass concentration at a certain time, t, and C0 is the initial biomass concentration.

#### 2.1.1. Dry Cell Weight Protocol

A millipore membrane filter (diameter 47 mm, μ = 0.45 μm) placed in an aluminum cup (ø x h = 70 x 6 mm) was dried 24 h in an oven and weighed. Note that 2–10 mL of culture was filtered through a dried filter. The filter was rinsed with sea water (2 x filtered volume). Finally, the filter + biomass was dried for 24 h and weighed again. The DCW was calculated as: [weight of (filter + aluminum cup + biomass)] − [weight of (filter + aluminum cup)]/L of filtered culture and expressed as g/L.

#### 2.1.2. Scale-Up in 2-L Photobioreactor

The optimized conditions were carried out in duplicate in 2-L photobioreactors (Applikon Schiedam, The Netherlands). All cultures were sparged continuously with air at flow rate of 0.5 L/min. The pH was controlled at 8 by automatic on-demand injection of 0.25 N of HNO_3_. Temperature was controlled at 20°C by circulating water. Light was supplied continuously with external light panels ranging from 70 to 300 μE m^−2^ s^−1^. The cultures were ensured to be axenic by checking the cellular morphology using 100x microscopy every day.

### 2.2. Photosynthesis and Respiration Measurements

#### 2.2.1. Chlorophyll Fluorescence

All the photosynthetic parameters were determined using a Speedzen MX fluorescence imaging setup (JBeamBio, France) as described in Vandystadt et al. (2009). For these experiments, the first version of the Speed Zen (which has no number) was used. In this setup, actinic light and saturating pulses are provided by green LEDs peaking at 520 nm, while measuring pulses (duration 250 ms) are provided by blue LEDs peaking at 470 nm. The detection time after every detecting pulse was 15 μs. No binning was applied.

For each sample, 3 x 200 μL of algal culture were transferred in a 96-well plate. Maximum quantum yield of PSII (Fv/Fm = (Fm - F0)/Fm) was determined after 15 min of dark incubation, where Fm and F0 are the maximum and minimum fluorescence of dark-adapted cells, respectively. Nonphotochemical quenching (NPQ) was calculated as (Fm-Fm')/Fm', where Fm' and Fm are maximum fluorescence of light-adapted and dark-adapted cells, respectively. NPQ was measured at 400 μE. This light intensity was chosen after preliminary experiments on light dependency of NPQ that showed 400 μE was enough to reach the maximum value, without inducing photodamage.

#### 2.2.2. Respiration Rates

Respiration rates were measured as O_2_ exchange rates using a Clark-type oxygen electrode at 19°C (Hansatech Instruments). The cells from the exponential phase of flask experiment were collected, at day 4, and the concentration adjusted to 3*10^7^ cells/mL with respective medium (i.e., E10: ESAW medium with 10 times extra N and P as described in section 2.1, E10+GLY: E10 supplemented with glycerol, EE: ESAW enriched medium developed in this study as described in section 3.2.1, EE+GLY: EE supplemented with glycerol, EE+BIC: EE supplemented with bicarbonate, EE+BIC+GLY: EE supplemented with bicarbonate and glycerol). The O_2_ in the medium was calculated in both light (i.e., photosynthesis) and dark (i.e., respiration). The light intensity used for the determination of O_2_ in the photosynthesis was 200 μE m^−2^ s^−1^, which was enough to reach the maximum value as per our preliminary experiments.

### 2.3. Metabolite Analysis

#### 2.3.1. Nitrogen and Phosphate Concentration

Nitrogen and phosphate concentration in supernatant were determining using a Merk RQflex reflectometer (E. Merck, 64271 Domsstadt, Germany) with test strips (Reflectoquant nitrate and phosphate).

#### 2.3.2. Glycerol Concentration

The glycerol concentration of 2 mL of filtered supernatant was measured with a Shimadzu HPLC with a Hi-plex H+ (7.7 X 300 mm) Agilent column. The analysis was performed using the mobile phase 5 mM H_2_SO_4_. The detection wavelength was set at 210 nm using a RI RID-10A Detector with a flow rate of 0.6 mL/min and a temperature of 60°C. The peak quantification was performed by comparison with a range of 6 solution standards.

#### 2.3.3. Total Lipids Extraction

Total lipids were extracted according to Folch et al. (1957). About 20 mg of dried cells were homogenized with 1 mL of chloroform/methanol 2:1. The cells were then lysed using a TissueLyser II (Qiagen) with an agitation of 1 min and a frequency of 300 s^−1^. The lysate was washed with 200 μL of NaCl 0.9 % and vortexed for some seconds in order to form the emulsion. The solution was centrifuged 5 min at 13,000 rpm to separate the two phase and the lower phase placed in fresh pre-weighed glass tubes. The upper phase was washed with chloroform; lysis and centrifugation steps were repeated in order to recover more lipids. The wash with chloroform was repeated at least twice. The lower phases (containing lipids) collected in glass tubes were evaporated under a nitrogen stream at 65°C. The glass tubes were weighed to determine the percentage of lipids extracted per dry cells.

#### 2.3.4. Fatty Acid Determination

For the fatty acid isolation, 1 mL of chloroform was added to 2 mg of freeze-dried biomass in 7-mL glass tubes. The tubes were heated in a shaking water bath at 80°C for 90 min. Note that 1.5 mL of ultra-pure water and 2 mL of heptane were added to the tubes, once cooled to the room temperature, and mixed by vortexing. The tubes were then centrifuged for 5 min at 3,000 rpm to separate the phase. The upper phase containing heptane and fatty acid methyl ester (FAMEs) was recovered in a labeled screw vial. Note that 10 mg of C23:0-Me was added to the samples and used as internal standard. Finally, the samples were injected in a gas chromatography coupled with a flame ionization detector (GC-FID) from Shimadzu and a 30 m x 0.25 mm x 0.25 μm DB-23 capillary column from Agilent. FAMEs were identified by GC-FID and compared with different standards (Supelco 37 component FAME Mix and PUFA No. 3, from Menhaden). The FAMEs were quantified through the comparison against the C23:0 internal standard.

#### 2.3.5. Pigment Determination

The pigment extraction was carried out under hood in the dark. Two to three milligram of freeze-dried biomass was dissolved in 1 mL of 95% ethanol/acetonitrile (60/40) in 2 mL “Safe Lock” eppendorf and stainless-steel beads were added. The tubes were ground in a TissueLyser II QUIAGEN for 1 min at 30 Hz s^−1^. The tubes were then placed in an ultrasonic water bath for 10 min and centrifuged at 13,000 rpm for 5 min. If the pellet was white, 900 μL of supernatant was placed in a labeled amber vial and 180 μL of water was added. Otherwise, the supernatant was collected in a separate tube, new solvent was added, and ultrasonication was repeated until the pellet was colorless. The pigment determination was carried out with a HPLC DIONEX Ultimate 3000 and a detector DAD 3000. The reverse phase was the column C8 Agilent Elipse XDB 3.5 μm (4.6 x 150 mm) with pre-column C8 XDB Agilent (4.6 x 12.4 mm). Solvent A consisted of ethanol 50%/28 mM tetra-butyl ammonium acetate at pH 6.5 (70/30) and solvent B of ethanol/ACN (60/40). The elution gradient was: 0 min: 95% A and 5% B; 3 min: 60% A and 40% B; 7 min: 60% A and 40% B; 21 min: 20% A and 80% B; 26 min: 20% A and 80% B; 27 min: 5% A and 95% B; 32 min: 5% A and 95% B; 37 min: 95% A and 5% B; 40 min: 95% A and 5% B. The flow rate was kept at 1 mL/min and the column was set at 60°C. Pigments were detected by diode-array spectroscopy (wavelength range: 280–800 nm) with comparison of the standards. For the identification and the quantification of the pigments the following standards were used; β-carotene (Fluka, ref.: 1448298), Fucoxanthin (Sigma-Aldrich, ref.: F6932-10MG) and a mix of pigments derived from the extract of a strains present in FERMENTALG culture collection.

#### 2.3.6. Carbohydrate Determination

For the carbohydrate determination, 250 μL of 80% sulfuric acid (H_2_SO_4_) was added to 2 mg of freeze-dried biomass in 7 mL screw cap glass tubes to hydrolyze samples. The tubes were centrifuged for 1 min at 3000 rpm and then heated in a dry bath at 110°C for about 1 min. The samples were placed for 15 min in an ultrasonic bath. Blank, standards, and samples were prepared in new glass tubes for the spectrophotometer analysis as follows: Blank: 15 μL of ultrapure water + 720 μL of 0.625% phenol + 1.5 mL of 80% H_2_SO_4_, standards: 15 μL of standards solution + 720 μL of 0.625% phenol + 1.5 mL of 80% H_2_SO_4_, sample: 15 μL of sample + 720 μL of 0.625% phenol + 1.5 mL of 80% H_2_SO_4_. The tubes were then vortexed and placed in a dry bath at 110°C for 5 min and finally the tubes were placed in an ice-water bath for 1 min to stop the reaction. Note that 150 μL of sample was then placed in a 96-well plate and put in a plate reader spectrophotometer to read the absorbance at 492 nm. Biomass concentration in the hydrolysis tube: [B] = (mg of dried biomass x 1000)/volume of H_2_SO_4_ 80% (250 μL). Quantity of biomass in the staining tube: QB = [B] (μg/μL) x the hydrolyzate collected (15 μL). Quantity of total carbohydrates in the tube: QC = OD 492/coefficient of calibration obtained from the standard. Concentration of carbohydrates in biomass: [carbohydrates] = (QC/QB) x 1000. The standards used to generate the calibration curve were serial dilution of a glucose stock solution (from 4 to 0.07 g).

### 2.4. Statistical Analyses

Growth profile Ln(C/C0) data were fitted by linear regression between day 1 and 6 corresponding to the exponential phase in each medium. The slopes of the obtained regression curves, corresponding to the growth rate, were then compared by *t*-test analysis using GraphPad 9.01. The nutrient consumption was compared in mixotrophy and phototrophy growth in the different medium using two-way analysis of variance (ANOVA). For all the others analysis, i.e., biomass and photosynthesis, mixotrophy, and phototrophy conditions were compared using one-way ANOVA. *P*-values were used to quantify the variability between control (phototrophy) and treatment (mixotrophy) under different cultivation conditions. Data were considered significant for *p*-values < 0.1.

### 2.5. Mathematical Modeling

#### 2.5.1. Genome-Scale Metabolic Model

A genome-scale metabolic model (GSM) of *P. tricornutum* developed in Singh et al. (2015) and Villanova et al. (2017), starting from the model of Hunt et al. (2014), was used in this study. The model consists of 450 reactions, 146 transporters (including external, inter-compartmental and biomass transporters), and 525 internal metabolites, and comprises cytosolic, plastidial, mitochondrial, and peroxisomal compartments. For this study, reactions were also balanced for proton and O_2_, in addition to all other atoms such as carbon, nitrogen, phosphorus, sulfur, and magnesium. It can use phosphate, sulfate, ammonium, nitrate, magnesium, and inorganic and/or organic carbon as input material for biomass production. It has been validated with respect to the laws of energy and mass conservation, and is able to produce all major biomass components (carbohydrate, lipid, amino acids, nucleotides, etc.) in phototrophic and mixotrophic conditions in experimentally observed proportions.

#### 2.5.2. Model Analysis: Linear Programming Formulation

Model analysis was undertaken using the linear programming (LP) approach based on the law of mass conservation (Fell and Small, 1986; Varma and Palsson, 1994). The minimization of total flux in the network, as a proxy for economy of investment in enzymatic machinery (Holzhütter, 2006; Poolman et al., 2009), was used as the objective function along with the steady-state assumption and additional constraints, as defined in the following equation:

(1) $$\begin{array}{l} \text{minimize }\begin{array}{ll} : & \left|\text{v}\right| \end{array}\\ \text{subject to}\{\begin{array}{l} \text{Nv}\text{ = }0\\ {\text{v}}_{\nu }\text{=}\nu \\ {\text{v}}_{txTAG}\text{ = }TAG\\ {\text{v}}_{txHCO3}\text{ = }HCO_{\text{3}}\\ {\text{v}}_{txGlycerol}\text{ = }Glycerol \end{array} \end{array}$$

where **v** is the vector of all reaction fluxes and **N** is the stoichiometry matrix; the objective is to minimize the sum of all (absolute) flux values (including transporters), subject to the constraints: **Nv** = **0** (the steady-state assumption), *v*_ν_ = ν which defines the photon flux (light) into the system, *v*_*txTAG*_ = *TAG* defines demand for TAG production, *v*_*txHCO*_3__ = *HCO*_3_ and *v*_*txGlycerol*_ = *Glycerol* define constraints on HCO_3_ and glycerol transporters. Analysis under phototrophic condition has light as the only source of energy and CO_2_, and HCO_3_ where stated, as inorganic carbon source and no source of organic carbon (i.e., *v*_*txGlycerol*_ = 0). Mixotrophic condition in this study refers to conditions with glycerol as organic carbon source. All computation was achieved using the ScrumPy metabolic modelling package (Poolman, 2006).

## 3. Results

### 3.1. Mathematical Modeling

#### 3.1.1. Model Analysis in Phototrophic and Mixotrophic Condition and the Effect of Bicarbonate

The LP in Equation (1) was solved for 1 unit of TAG demand (*v*_*txTAG*_ = 1.0) under four conditions: (i) +BIC+GLY: mixotrophic condition in the presence of HCO_3_ (i.e., no constraint is set on glycerol and HCO_3_ transporters), (ii) –BIC+GLY: mixotrophic condition in the absence of HCO_3_ (*v*_*txHCO*_3__ = 0 while no constraint is set on glycerol transporters), (iii) +BIC-GLY: phototrophic condition in the presence of HCO_3_ (i.e., *v*_*txGlycerol*_ = 0 while no constraint is set on HCO_3_ transporters) and, (iv) –BIC-GLY: phototrophic condition in the absence of HCO_3_ (*v*_*txHCO*3_ = *v*_*txGlycerol*_ = 0).

The model is able to produce TAG under all four conditions; however, the objective value along with net consumption and production of substrates vary under these conditions and is summarized in Tables 1, 2, respectively. Availability of glycerol and/or HCO_3_ reduces the objective value and the requirement of light as energy source.

**Table 1:** Objective value and number of reactions in linear programming solution for each condition.

	**+BIC+GLY**	**–BIC+GLY**	**+BIC–GLY**	**–BIC–GLY**
No. of reactions	69	103	67	95
Objective value	1.26 × 10^3^	1.63 × 10^3^	2.12 × 10^3^	2.93 × 10^3^

**Table 2:** Net production and consumption of substrates (net stoichiometry of linear programming solution) for each condition.

	**+BIC+GLY**	**–BIC+GLY**	**+BIC–GLY**	**–BIC–GLY**
x_WATER	26.857	48.676	−32.0	−47.99
x_CARBON-DIOXIDE	9.142	21.507	−35.0	−50.999
x_HCO3	−16.0	0.0	−16.0	0.0
x_GLYCEROL	−14.714	−24.169	0.0	0.0
x_TAG	1	1	1	1
x_Photon	−360.333	−232.676	−695.667	−924.857
x_OXYGEN-MOLECULE	21	−12.091	72.5	72.5
x_PROTON	−15.999	0.0	−16.0	0.0

*Here, negative value denotes consumption while positive value denotes production of substrates*.

Glycerol, when available, is the preferred carbon source for lipid production. It enters metabolism through conversion to (i) glycerol phosphate, by glycerol kinase, which provides the immediate glycerol backbone for TAG synthesis, and (ii) DHAP, by glycerol-3-phosphate dehydrogenase, which enters the central carbon metabolism through glycolysis and cytosolic PPP. On the contrary, under phototrophic condition (in the absence of glycerol), carbon demand is met through inorganic carbon fixation through the Calvin cycle for which the enzymatic cost (objective value) and the requirement for light energy are higher compared to mixotrophic conditions, as shown in Tables 1, 2, respectively.

Bicarbonate, when available, is utilized by PEP/pyruvate carboxylase for anaplerotic production of C4 moieties from C3 moieties. The C3 skeleton (pyruvate and/or PEP) is produced through glycolysis under mixotrophic conditions and the Calvin cycle followed by lower glycolysis under phototrophic conditions. Anaplerotic fixation of HCO_3_ contributes one-third of carbon demand under both conditions (Table 2). Interestingly, in the absence of HCO_3_, photorespiration is active under both conditions. Glycolate produced through photorespiration is converted to glyoxylate, by glycolate dehydrogenase, that then enters the glyoxylate shunt, along with acetyl-CoA produced through pyruvate dehydrogenase, to produce C4 moieties. This route for production of C4 moieties is pricey, compared to anaplerotic HCO_3_ fixation, in terms of enzymatic cost and carbon loss under mixotrophic condition, as reflected by the objective value and net stoichiometry in Tables 1, 2, respectively. Thus, mixotrophic condition with HCO_3_ (i.e., +BIC+GLY) was regarded as the optimal condition for lipid production.

#### 3.1.2. Lipid Demand Variation Analysis

To further investigate the response of the system to varying lipid demand under mixotrophic condition in the presence of HCO_3_, LP in Equation (1) was solved repeatedly with incremented demand for lipid production, *v*_txTAG_, until no feasible solution was possible, as described in Poolman et al. (2009, 2013) and Villanova et al. (2017). An upper limit was set on glycerol uptake (*v*_*txGlycerol*_ ≤ *maxG*) and photon flux (*v*_ν_ ≤ *maxP*) as a proxy for maximum glycerol uptake and light limitation, respectively, and on rubisco reaction (*v*_Carboxylase_ + *v*_Oxygenase_ ≤ *maxC*) as proxy for saturation of the Calvin cycle, as in Villanova et al. (2017).

**Figure 1:**
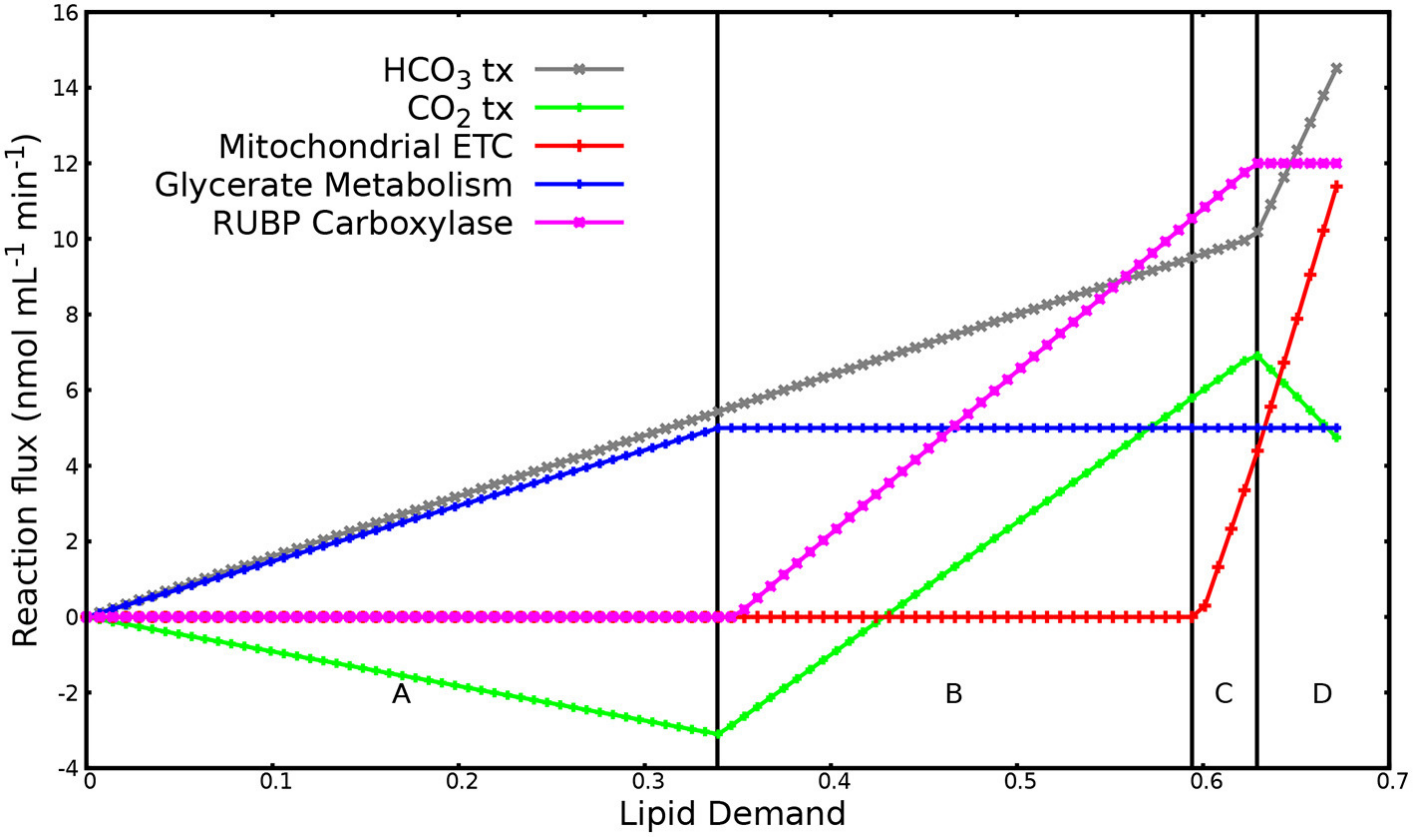
Reaction responses to increase in lipid demand. The plot has been divided into four major regions labeled A to D based on the flux patterns. Region A: reaction response when flux in glycerol uptake, photon flux, and rubisco reaction is not saturated. Region B: reaction response when flux in glycerol transporter saturates. Region C: reaction response when photon flux saturates. Region D: reaction response when flux in Rubisco reaction saturates. Negative flux denotes export of substrate out of the system while positive flux denotes import of substrate into the system.

A total of 108 reactions showed variation over the range of imposed lipid demand. Figure 1 shows the reaction responses to increase in lipid demand. Other reactions varying over the lipid demand followed one or other pattern in Figure 1. The change in the metabolic response (regions A, B, C, and D) adjusted with the saturation of glycerol uptake, photon flux, and flux in rubisco reaction. Region A marks low lipid demand and is solely met through glycerol uptake and anaplerotic HCO_3_ fixation. In region B, flux in glycerol uptake saturates because of the imposed upper limit. The increasing demand for carbon is met through increased flux in CO_2_ fixation through the Calvin cycle and anaplerotic HCO_3_ fixation. In region C, flux in photon uptake saturates. Thus, the increased energy demand for increasing lipid production is met through increased flux in mitochondrial electron transport chain. In region D, flux in rubisco reaction saturates. The increasing demand for carbon is met through increased flux in anaplerotic HCO_3_ fixation through PEP/pyruvate carboxylase. This region also marks the production of maximum O_2_ (≈ 31 nmol/mL/min), which is in range of experimentally observed O_2_ evolution under mixotrophic condition with HCO_3_, as shown in **Figure 4** (1 nmol/mL/min ≈ 0.004–0.0085 nmol/mg Chl/min). Overall, the optimal metabolic state to meet the high lipid demand utilizes glycerol metabolism, light reactions, mitochondrial electron transport chain, the Calvin cycle, and anaplerotic HCO_3_ fixation.

### 3.2. Experimental Analysis

#### 3.2.1. Optimization of the ESAW Medium

Elemental composition of E10 medium was optimized based on the elemental balancing of biomass and medium composition. This is a powerful method to improve the biomass yield of microalgae as shown in other strains (Mandalam and Palsson, 1998; Takács et al., 2007; Harun et al., 2010). This analysis revealed that E10 is not only deficient in macronutrients (i.e., nitrogen and phosphorus) but mostly in micronutrients (i.e., iron, manganese, zinc, and copper). Thus, a new medium (called ESAW enriched [EE]) was redesigned by increasing the concentration of NaNO_3_, NaH_2_PO_4_.H_2_O, FeEDTA, ZnSO_4_.7H_2_O, MnSO_4_.4H_2_O, and CuSO_4_. 5H_2_O to 2.5 g/L, 0.20 g/L, 0.0216 g/L, 0.589 mg/L, 2.55 mg/L, and 0.66 mg/L, respectively. The amount of other elements were maintained the same as E10 medium. EE composition is provided in Supplementary File 2.

Based on the model observations in section 3.1, the EE medium under phototrophic and mixotrophic (with glycerol as organic carbon source) condition was supplemented with NaHCO_3_ (1.26 g/L) to improve the algal growth capacity (i.e., EE+BIC: EE supplemented with bicarbonate and EE+BIC+GLY: EE supplemented with bicarbonate and glycerol, respectively).

#### 3.2.2. Effect of Glycerol, Bicarbonate, and Nutrients on Phaeodactylum Biomass at Small Scale

**Figure 2:**
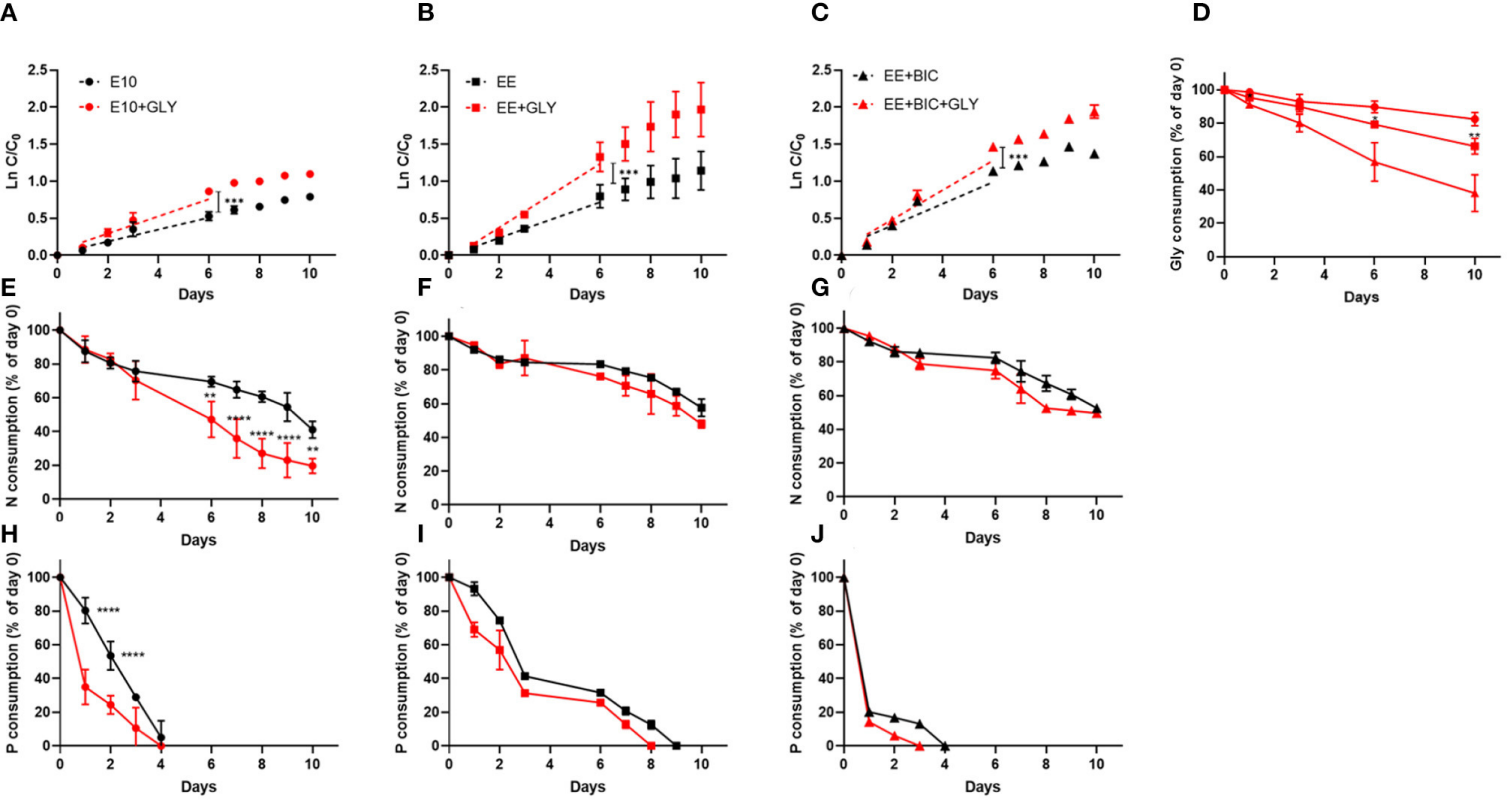
Growth curve, glycerol, nitrate, and phosphate consumption in the initial medium E10 (circle), optimized medium EE (square), and EE+BIC (triangle) in phototrophy (black) and in mixotrophy (red) conditions. Log plot of growth curves in **(A)** E10, **(B)** EE, **(C)** EE+BIC in phototrophy and mixotrophy conditions. C is the biomass concentration expressed in g/L at any time and C0 is the initial biomass concentration. Dotted lines represent the regression curve during the exponential phase of growth (1–6 days). The t-test performed on the slopes were significant with *p*-value (***p < 0.001). **(D)** Glycerol consumption in E10+GLY, EE+GLY, and EE+BIC+GLY. Nitrate consumption expressed as % of day 0 in **(E)** E10, **(F)** EE, **(G)** EE+BIC and phosphate consumption in **(H)** E10, **(I)** EE, and **(J)** EE+BIC. The nutrient consumption was compared in mixotrophy and phototrophy growth in the different medium using two-way ANOVA. Data were considered significant for *p*-values(****P < 0.0001) and (**p < 0.01). Each point expressed as mean ± stdev (*n* = 4) except for +BIC treatments where *n* = 2. E10, ESAW 10XN,P; EE, ESAW enriched; EE+BIC, ESAW enriched + bicarbonate; GLY, glycerol.

During the exponential phase (1–6 days), the growth rate was significantly increased in mixotrophy compared to phototrophy in each medium (*p* < 0.001) (Figures 2A–C). The exponential growth rate of each condition, the results of *t*-test analysis, and final biomass concentration are shown in Table 3. In addition, EE medium, described in section 3.2.1, in 50-mL Erlenmeyer flasks, increased growth rate by a factor of 1.85 and 1.5 under mixotrophic and phototrophic regime, respectively, as compared to initial E10 medium, without changing the cell dimension. The enhanced mixotrophic growth observed with EE was also mainly due to the positive effect caused by the trace elements (Supplementary Figure 1). The positive effect of the EE medium on mixotrophic growth became even larger upon the addition of bicarbonate in the medium increasing both growth and glycerol consumption (Figures 2C,D and Table 3).

**Table 3:** Exponential growth rate, *t*-test analysis, and final biomass concentration in the different media.

**Condition**	**Exponential growth rate (d^**−1**^)**	***T*****-test analysis**		**Final biomass concentration (g/L)**
		**Phototrophy vs. Mixotrophy**	**Media vs. Improved media**	
E10	0.08 ± 0.02	E10 vs. E10+GLY (****)	E10 vs. EE (****)	0.874 ± 0.075
E10+GLY	0.116 ± 0.04		E10+GLY vs. EE+GLY (****)	1.177 ± 0.059
EE	0.121 ± 0.008	EE vs. EE+GLY (****)	EE vs. EE+BIC (*)	1.095 ± 0.027
EE+GLY	0.215 ± 0.05		EE+GLY vs. EE+BIC+GLY (ns)	1.857 ± 0.104
EE+BIC	0.147 ± 0.013 (*n* = 2)	EE+BIC vs. EE+BIC+GLY (****)		1.306 ± 0.117
EE+BIC+GLY	0.2 ± 0.08 (*n* = 2)			2.017 ± 0.032

Growth rate are expressed as mean ± standard deviation with n = 4 unless otherwise stated. Data were considered significantly different for p-value <0.1 (*) and <0.0001(****), not significant (ns): p-value >0.1.

Nitrate and phosphate consumption were also compared in mixotrophic and phototrophy in the different medium (Figures 2E–J). Only in the E10 medium, characterized by lower initial nitrate and phosphate concentration then EE, the consumption of these nutrients was significantly (*p*-value < 0.01) enhanced in mixotrophy (Figures 2E,H). Phosphate is consumed very rapidly but this does not arrest the growth rate probably because of the polyphosphate accumulation mechanism (Martin et al., 2014; Abida et al., 2015).

**Figure 3:**
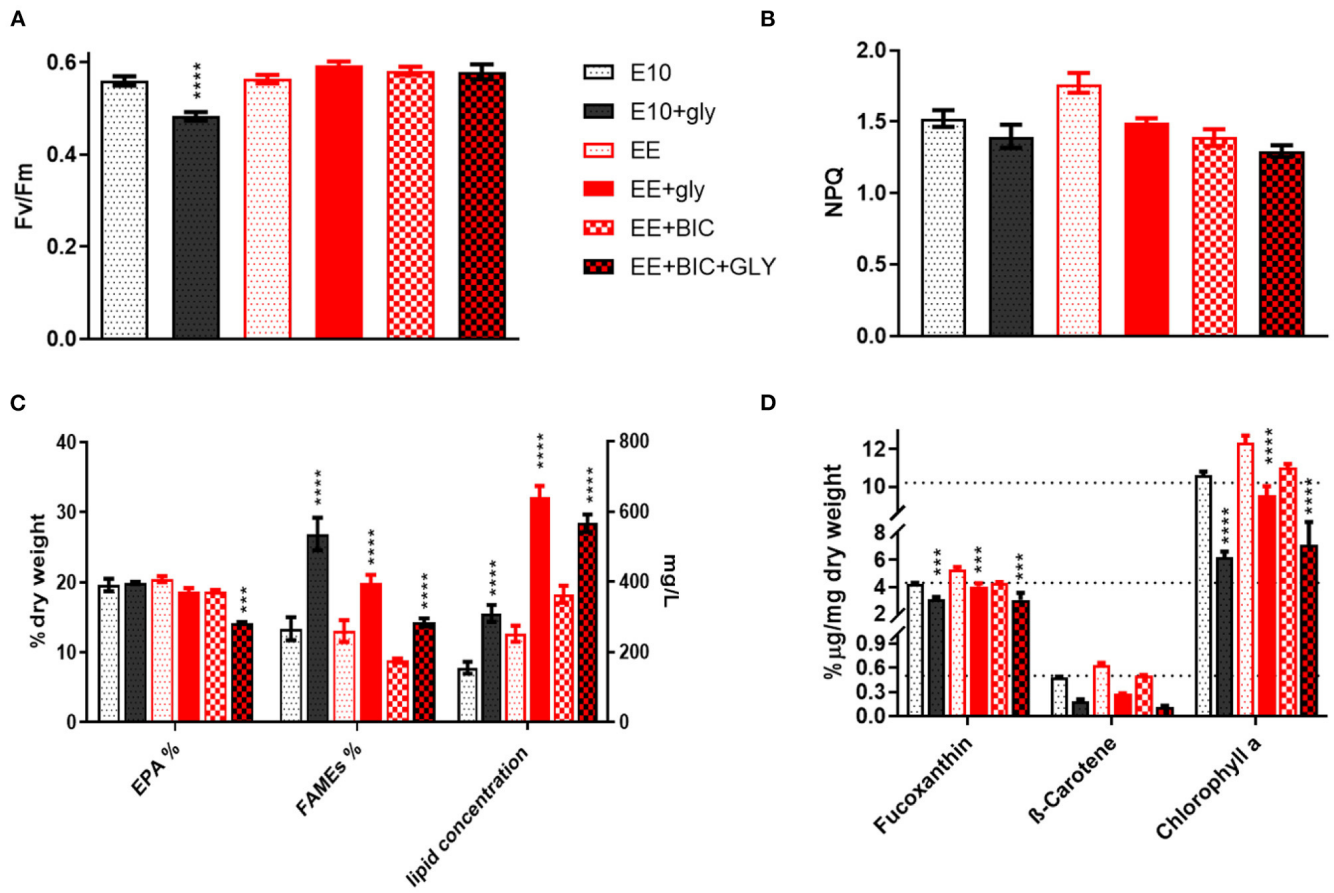
Biomass analysis and photosynthetic parameters analysis. **(A)** Fv/Fm and **(B)** NPQ measurement, **(C)** Lipids, and **(D)** pigments analysis in the initial medium E10 and in the optimized medium EE and EE+BIC in both mixotrophy and phototrophy. In **(C)**, right Y-axis (mg/L) is for lipid concentration while the left Y-axis(%) is for FAMES and EPA. One-way analysis of variance (ANOVA) test was applied in order to compare the phototrophy and mixotrophy in different media. Data were considered significant for *p*-values (****p < 0.0001) and (***p < 0.001). Each point expressed as mean ± stdev (*n* = 4). E10, ESAW 10XN,P; EE, ESAW enriched; EE+BIC, ESAW enriched + bicarbonate; GLY, glycerol.

On the last day (i.e., day 10) of the experiment, the biomass was surveyed combining photosynthesis, pigment, and lipid analysis (Figures 3A–D). The photosynthesis performances and the photoprotection mechanisms were studied by the determination of the parameters Fv/Fm and NPQ, respectively (as described in section 2.2.1). The data showed that the glycerol decreases the photosynthesis performance only in E10 (*p*-value < 0.0001), most likely due to the rapid consumption of the N (Figures 2D, 3A). No significant differences were detected in photoprotection in the tested conditions (Figure 3B).

The lipid analysis was divided into eicosapentaenoic acid (EPA), fatty acid, and total lipid concentration (Figure 3C). The EPA concentration was similar in all the tested conditions with the exception of EE+BIC+GLY where the concentration was lower (*p*-value < 0.001). The glycerol enhanced the fatty acid content compared to their phototrophic counterpart (*p*-value < 0.0001) and its effect was higher in E10+GLY, possibly due to N starvation that trigger lipid production. In EE, which had higher nitrogen concentration, the glycerol mostly increased the total lipid concentration (Figure 3C, *p*-value < 0.0001). In addition, the bicarbonate increased the total lipids concentration, *p*-value < 0.0001, when compared to E10 and EE, under phototrophic conditions.

EE also increased the concentration of pigments, especially fucoxanthin, and chlorophyll A, as shown in Figure 3D, compared to E10 in both phototrophy and mixotrophy conditions with *p*-value < 0.01–0.0001. The mixotrophy, however, decreased the concentration of pigments in all the conditions as compared to their phototrophic counterparts with *p*-value < 0.001–0.0001, but this is less evident in EE+GLY that possess a higher concentration of nitrate and phosphate on the day of the analysis (i.e., day 10; Figures 2C,D). No statistically significant difference was observed in β-carotene under different conditions. However, decrease of carotenoids and chlorophyll in mixotrophy has been shown in *P. tricornutum* (Liu et al., 2009b), but also the nutrients limitation has a key role in the degradation of pigments (Alipanah et al., 2015, 2018). Data for all flask experiments are available in Supplementary File 3.

**Figure 4:**
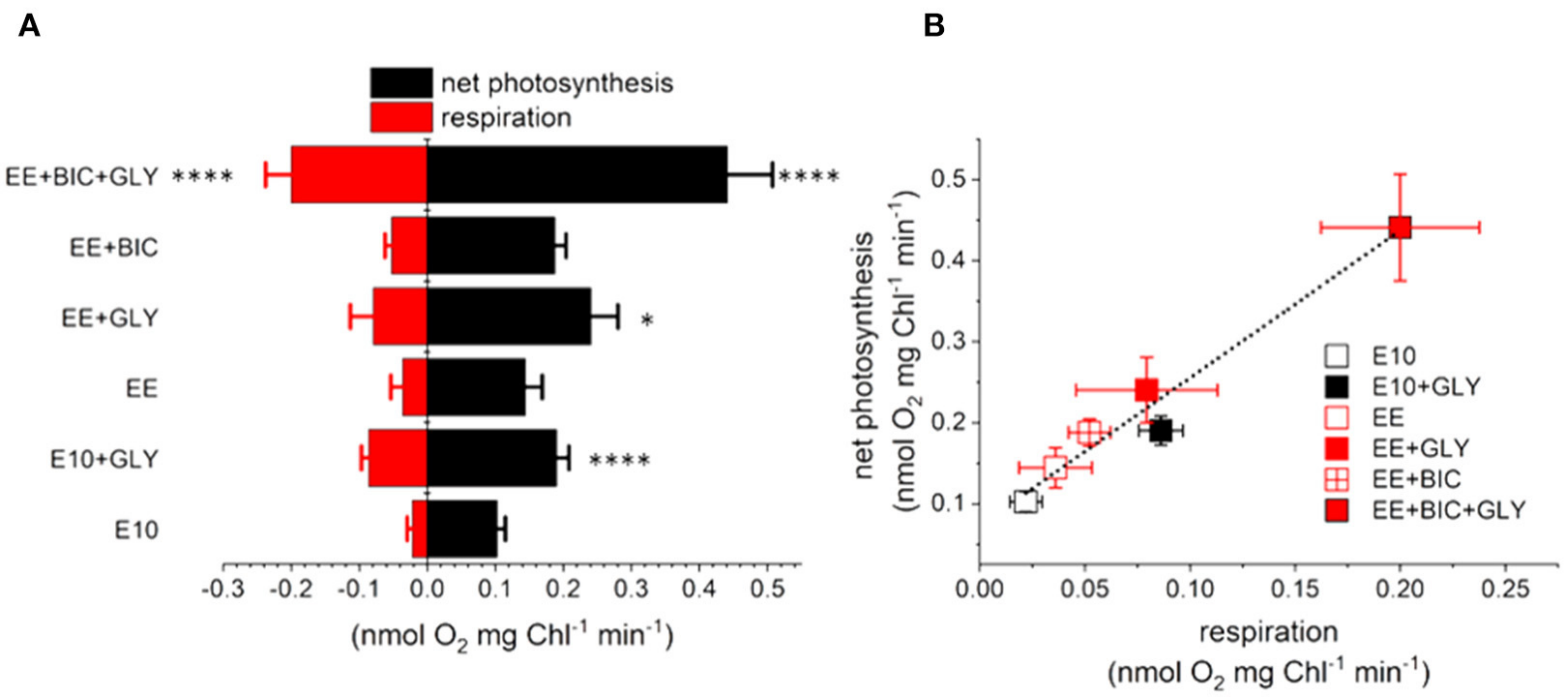
**(A)** Direct assessment of oxygen consumption/production by a polarographic approach in E10, EE, and EE + BIC in both phototrophy and mixotrophy (+GLY). Measurements were done in the light (i.e., photosynthesis, black) and in the dark (i.e., respiration, red). One-way analysis of variance (ANOVA) test was applied in order to compare the phototrophy and mixotrophy in different media. Data were considered significant for *p*-values < 0.1 (****p < 0.0001, *p < 0.1). **(B)** Relationship between oxygen uptake and net photosynthesis measured with a Clark electrode. Dotted lines represent a fitted regression. Each point expressed as mean ± stdev (*n* = 4–12).

Finally, respiration and photosynthesis rates were measured as O_2_ exchange rates using a Clark-type oxygen electrode with an *in vivo* experiment at 19°C (Hansatech Instruments) (as described in section 2.2.2). As shown in Figure 4A, the glycerol enhanced the respiration rates in all the tested conditions (*p*-value < 0.1–0.0001), confirming previous results (Grama et al., 2015). The oxygen consumption was higher in the optimized medium (i.e., both EE and EE+BIC), as expected based on the finding that glycerol consumption and growth performances are higher in these conditions (Figure 2). Net photosynthesis (calculated as oxygen evolution corrected by dark respiration) was also increased by glycerol, in a way that was commensurate to the respiration enhancement (Figure 4B). Indeed, we found a linear relationship between the photosynthetic and respiratory performances. Overall these results suggest that mixotrophy enhances respiration (via glycerol consumption) and photosynthesis, possibly through energetic exchanges between the two energy making organelles in line with earlier hypotheses (Bailleul et al., 2015).

#### 3.2.3. Scale-Up in 2-L Photobioreactor

The experiment was further upscaled to 2-L photobioreactors, as specified in section 2.1.2, in E10, EE, and EE+bicarbonate under both phototrophic and mixotrophic condition. Photobioreactors with mixotrophic culture and EE + BIC condition were supplemented with 4.6 and 1.26 g/L of glycerol and NaHCO_3_, respectively, at a regular time interval. Since HNO_3_ was also used to regulate the pH of the culture, as mentioned in section 2.1.2, the cultures were never deficient in terms of nitrogen while NaH_2_PO_4_ was added to the culture as needed. However, in the samples E10 and E10+GLY the pH was regulated by the addition of 0.4 N of H_2_SO_4_ to maintain the original N concentration of the medium.

**Figure 5:**
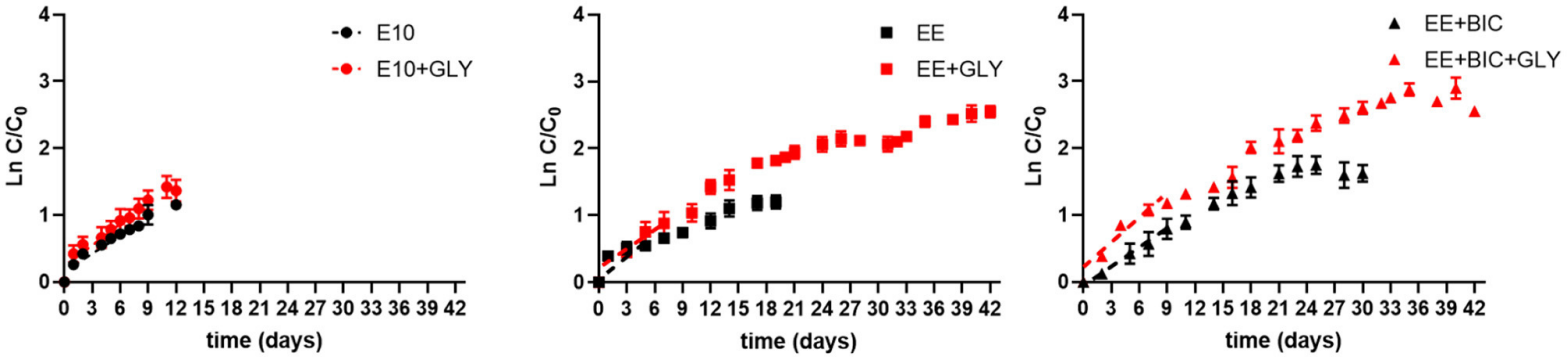
Scale-up to photobioreactors. Log plot of growth curves in 2-L fermentor in E10 (circle), EE (square), and EE+ BIC (triangle) in both phototrophic (black) and mixotrophic (red) modes. C is the biomass concentration expressed in g/L at any time, and C0 is the initial biomass concentration. Dotted lines represent the regression curve during the exponential phase of growth (1–6 days). The slopes of the curve (growth rates) of each media under phototrophy and mixotrophy conditions are shown in Table 4. Results are expressed as mean±stdev with n values as stated in Table 4.

As shown in Table 4 and Figure 5, exponential growth rate was largely increased in optimized medium (i.e., EE and EE+BIC) under mixotrophic condition when compared to initial conditions (E10). The growth rates in flask experiments (section 3.2.2 and Table 3 and bioreactor (Table 4) were highly comparable for equivalent conditions, and showed the same pattern of effects for medium supplementation and mixotrophy. The biomass concentration and biomass, lipid (as fatty acids and EPA), carbohydrates, and fucoxanthin productivities were compared in the different conditions and are summarized in Table 4. EE+BIC+GLY proves to be the best condition. The biomass concentration is increased by a factor of about 9 comparing to the initial conditions E10. Pigment, fatty acid, EPA, and carbohydrate also increases with *p*-value < 0.0001 (Supplementary File 4), when compared to initial conditions (E10). Higher lipids, especially fatty acids, were shown in E10±GLY compared to EE±GLY, most likely because of the N limitation in E10 media that triggers lipid storage. Our result demonstrates that the light and supplementation of bicarbonate induced higher production of lipids even without N limitation overcoming the trade-off between growth and lipid production.

**Table 4:** Growth rate, final biomass concentration, maximum FAMEs, EPA, fucoxanthin, and carbohydrate productivity in the different media, E10, EE, and EE+BIC under phototrophic and mixotrophic conditions in 2-L photobioreactor.

	**E10**	**E10+GLY**	**EE**	**EE+GLY**	**EE+BIC**	**EE+BIC+GLY**
Growth rate, (d^−1^)	0.078 ± 0.004 (*n* = 5)	0.098 ± 0.004 (*n* = 6)	0.06 ± 0.01 (*n* = 6)	0.19 ± 0.02	0.16 ± 0.02 (*n* = 6)	0.18 ± 0.03 (*n* = 5)
Final biomass conc, (g/L)	1.32 ± 0.08 (*n* = 6)	1.46 ± 0.08 (*n* = 6)	1.6 ± 0.14 (*n* = 6)	5.03 ± 0.19 (*n* = 6)	2.58 ± 0.15 (*n* = 6)	11.55 ± 0.24
max FAMEs, (mg/L/d)	14.59 ± 1.12	32.45 ± 2.22	9.98 ± 1.78	23.80 ± 2.86	21.61 ± 3.39	51.96 ± 0.61
max EPA, (mg/L/d)	2.12 ± 0.08	2.87 ± 0.21	1.40 ± 0.14	3.98 ± 0.51	3.2 ± 1.7	9.51 ± 0.13
max Fucoxanthine, (mg/L/d)	0.36 ± 0.05	0.21 ± 0.11	–	–	0.71 ± 0.06	1.97 ± 0.34
max Carbohydrate, (mg/L/d)	16.76 ± 1.35	31.03 ± 2.61	4.95 ± 0.54	25.50 ± 2.06	16.85 ± 13.18	54.91 ± 2.40

*Results are expressed as mean ± stdev with n = 4 unless otherwise stated*.

## 4. Discussion and Conclusion

In this study, we have combined metabolic modeling and experimental approach to design an optimized growth media (called EE) for *P. tricornutum*. The model analysis suggested contribution of glycerol and HCO_3_ fixation toward lipid production. Further, it suggest that high lipid production utilizes various aspects of metabolism such as photosynthesis, respiration, inorganic (HCO_3_), and organic (glycerol) carbon fixation. The addition of both organic (i.e., glycerol) and inorganic C (i.e., NaHCO_3_), along with the optimization of the trace elements, improved both biomass quantity and quality in *P. tricornutum* in our study.

Carbon is the major macronutrient that affects the growth of microalgae and to increase the biomass/growth and storage compounds such as lipid and carbohydrate, it is necessary to increase the dissolved inorganic carbon (DIC) concentration, as shown in different algal species, including *Phaeodactylum* (Levering et al., 2016; Hammer et al., 2019). The most common practice to supplement the algal culture with the DIC is to bubble air into the growth medium. Another method includes addition of HCO_3_ to the media (Lohman et al., 2015; Mokashi et al., 2016), which is a cheaper and more suitable inorganic carbon alternative to the CO_2_. Diatoms, including *Phaeodactylum*, possesses biophysical and/or biochemical CO_2_ - concentrating mechanism (CCM) (Reinfelder et al., 2000; Roberts et al., 2007; Hopkinson et al., 2011; Matsuda et al., 2011). Biophysical CCMs involve active transport of CO_2_ or HCO_3_ and CA maintains equilibrium between the two species by catalyzing the reversible interconversion of CO_2_ and water into HCO_3_ and protons. Biochemical CCMs involving C4-type photosynthesis, on the other hand, utilizes carboxylation enzymes such as PEP/pyruvate carboxylase, which catalyzes the carboxylation of PEP/pyruvate with HCO_3_, forming a C4 carbon compound. This compound is then cleaved by decarboxylating enzymes to produce CO_2_ in the proximity of Rubisco (Sage, 2004). In addition, anaplerotic production of C4 skeletons through HCO_3_ fixation by PEP/pyruvate carboxylase independent of photosynthesis, as observed in the model (section 3.1), has also been reported in diatoms (Granum and Myklestad, 1999; Needoba and Harrison, 2004). Results in this study confirm that addition of HCO_3_ improves the algal growth, although further investigation using C labeling and multi-omics techniques would be required to confirm the fate and mechanism of HCO_3_ utilization in *P. tricornutum*.

In addition, most microalgae can simultaneously assimilate organic carbon such as glycerol, fructose, glucose, lactose, mannose, and acetate (Garcì et al., 2004; Villanova et al., 2017). *P. tricornutum* is also able to use organic carbon in presence of light, and in particular, the glycerol has been shown to be the best candidate for enhancing biomass and lipids productivity (Garcìa et al., 2005, 2006, 2013; Villanova et al., 2017). Model analysis shows that glycerol enters the central carbon metabolism through glycolysis and PPP and that both the Calvin cycle located in plastid and PPP located in cytosol can be active simultaneously. In other photosynthetic eukaryotes where both the Calvin cycle and PPP are located in the same compartment (i.e., plastid), the simultaneous operation of the Calvin cycle and oxidative limb of PPP would lead to the futile cycling of NADPH. This is prevented by the action of the thioredoxin system (Anderson, 1981, 1986; Sibley and Anderson, 1989; Schurmann and Jacquot, 2000). This is a redox mediated system that serves (among other things) to activate the oxidative limb of the PPP in the dark, and inactivate it in the light. Although diatoms possess the thioredoxin system, its targets are unclear: the only Calvin cycle enzyme under thioredoxin control appears to be fructose bisphosphate aldolase (Wilhelm et al., 2006; Kroth et al., 2008). Moreover, PPP is translocated to the cytosol. Overall, we propose that this compartmental re-arrangement and redox deregulation could possibly be a metabolic advantage for *P. tricornutum* to simultaneously activate both the processes.

The increased concentration of micronutrient has also been shown to enhance both biomass and biotech relevant molecules, as TAGs, probably due their involvement in the key enzymes of photosynthesis, respiration, and carbon fixation in microalgae (Morel et al., 2003). Here, we showed that the addition of both organic (i.e., glycerol) and inorganic carbon (i.e., NaHCO_3_), along with increased micronutrients (trace elements), improved both biomass quantity and quality in *P. tricornutum*. It also enhanced both the respiration and photosynthesis performance (both in the model analysis and experimental results), suggesting that there is an energetic coupling between chloroplast and mitochondria and the communication between the two organelles is crucial for optimizing carbon fixation and growth as reported by Bailleul et al. (2015).

The medium optimization by implementing the micronutrients and NaHCO_3_ supply largely enhanced mixotrophy growth, allowing to reach the state of the art biomass and lipid concentration levels. The total lipid and biomass concentration in our improved EE medium, under mixotrophic regime in the flask experiments, are higher (641 mg/L and 1.8 g/L, respectively) as compared to previous experiments by Yang et al. (2017) and Yodsuwan et al. (2017) in f/2 medium, which obtained total lipid and biomass concentration in the range of 40–133 mg/L and 0.2–0.4 g/L, respectively, for the same or higher duration of the cultivation time. Fucoxanthin concentration in EE medium is comparable to that in f/2 medium (4.47 mg/g DW) (Yang et al., 2017). In upscaled 2-L photobioreactor, Fucoxanthin concentration was increased by a factor of about 6 compared to the initial condition. The biomass concentration, which was achieved in the presence of relatively low light intensities (in the range of 70–300 μE m^−2^ s^−1^) is comparable to previous experiments by Garcìa et al. (2013) that used light intensity of 750 μE m^−2^ s^−1^ where similar biomass concentration was obtained, in comparable cultivation time, in both phototrophy and mixotrophy (5 and 14 g/L, respectively). A further investigation would be required to comment if increasing light intensity, as in Garcìa et al. (2013), would have further enhanced the biomass and lipid concentration in our improved media.

The addition of glycerol also enhances carbohydrates concentration in *P. tricornutum* (Villanova et al., 2017). Here, the carbohydrate productivity was enhanced of about 3 times compared to the initial condition. The inhibition of the biosynthesis of storage carbohydrates could potentially direct the carbon (derived from glycerol) toward TAG production as already reported in the case of the main sugar storage polymer, chrysolaminarin in Daboussi et al. (2014). Our study demonstrated that the combination of different optimization processes, i.e., elemental balancing, process design and mathematical model, can be successfully integrated to design an optimized growth media that, in our experiments, have increased the algal production capabilities. Moreover, the algal productivity and lipid production could be further enhanced by metabolic engineering and improving the quality and quantity of light.

## Data Availability Statement

The datasets presented in this study can be found in online repositories. GSM is available from BioModels repository with model ID MODEL2102080001 (www.ebi.ac.uk/biomodels/). GSM (in ScrumPy and SBML format) along with python scripts to generate the results reported in this study is available from https://gitlab.com/singhdi/phaeomodel.

## Author Contributions

VV, DS, GF, JP, DF, AL, and MP conceived and designed the experiments, and analyzed data. VV and DS performed experiments and wrote the manuscript. DS and MP performed the model simulation. All the authors approved the final version of the manuscript before the submission.

## Conflict of Interest

VV was employed by company Fermentalg SA. The remaining authors declare that the research was conducted in the absence of any commercial or financial relationships that could be construed as a potential conflict of interest.

## Supplementary Material

Supplementary File 1 Calibration curve obtained by the correlation of the absorbance and dry weight.

Supplementary File 2 EE medium composition.

Supplementary File 3 All experimental data and statistical analysis for flask experiments.

Supplementary File 4 All experimental data and statistical analysis for 2-L photobioreactor experiments.

Supplementary Figure 1 Optimization of growth medium in mixotrophic condition. **(A)** Growth curve (from single experiment) and **(B)** pictures of flasks in the last day of cultures in the initial medium E10 (black line) in E10+N,P (green line), E10+Fe (yellow line), E10+Me (blue line), and in the optimized medium EE (red line). E10, ESAW 10XN,P; E10+ N,P, ESAW 10XN,P + enriched concentration of N and P; E10+ Fe, ESAW 10XN,P + enriched concentration of Fe; E10+Me, ESAW 10XN,P + enriched concentration of Cu, Zn, and Mn; EE, ESAW enriched.
