# Supplementary material for: Boosting Biomass Quantity and Quality by Improved Mixotrophic Culture of the Diatom Phaeodactylum tricornutum
Source: Front Plant Sci. 2021 Apr 9;12:642199. doi: 10.3389/fpls.2021.642199 (PMC8063856; doi:10.3389/fpls.2021.642199)
Supplement: Supplementary Figure 1 — Optimization of growth medium in mixotrophic condition. (A) Growth curve (from single experiment) and (B) pictures of flasks in the last day of cultures in the initial medium E10 (black line) in E10+N,P (green line), E10+Fe (yellow line), E10+Me (blue line), and in the optimized medium EE (red line). E10, ESAW 10XN,P; E10+ N,P, ESAW 10XN,P + enriched concentration of N and P; E10+ Fe, ESAW 10XN,P + enriched concentration of Fe; E10+Me, ESAW 10XN,P + enriched concentration of Cu, Zn, and Mn; EE, ESAW enriched. [file Image_1.pdf]

Supplementary Material

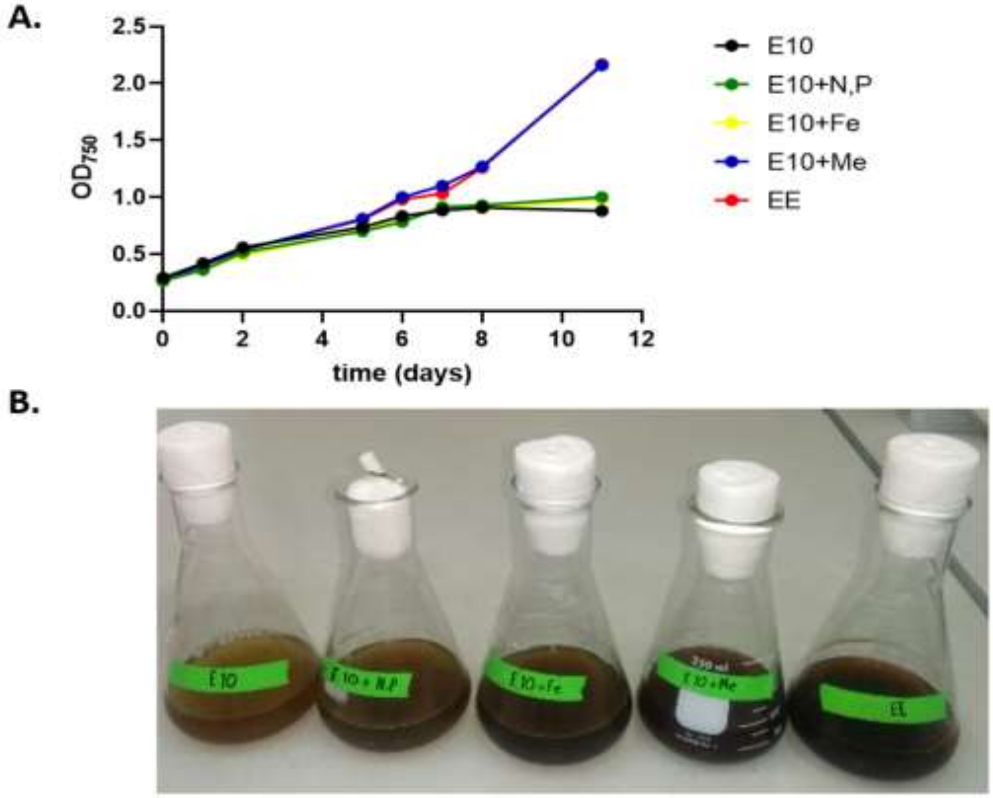

Supplementary Figure 1. Optimization of growth medium in mixotrophic condition. A. Growth curve (from single experiment) and B. pictures of flasks in the last day of cultures in the initial medium E10 (black line) in E10+N,P (green line), E10+Fe (yellow line), E10+Me (blue line), and in the optimized medium EE (red line). E10 = ESAW 10XN,P; E10+ N,P = ESAW 10XN,P + enriched concentration of N and P, E10+ Fe = ESAW 10XN,P + enriched concentration of Fe, E10+Me = ESAW 10XN,P + enriched concentration of Cu, Zn and Mn, EE = ESAW enriched.
